# From MS1 to Structure: A Van Krevelen–DBE–Aromaticity‐Based Framework for Annotating Specialized Metabolites via High‐Resolution Mass Spectrometry

**DOI:** 10.1002/rcm.10145

**Published:** 2025-09-22

**Authors:** Nerilson M. Lima, Luana A. Pereira, Lucas S. Tironi, Matheus P. G. do Carmo, Milbya L. Costa, Renato A. Oliveira, Salva Asghar, Vinicius Fortes da Silva

**Affiliations:** ^1^ Institute of Chemistry Federal University of Alfenas Alfenas Minas Gerais Brazil

**Keywords:** double bond equivalent (DBE), high‐resolution mass spectrometry (HRMS), natural products, untargeted metabolomics, Van Krevelen diagram

## Abstract

**Rationale:**

Classifying specialized metabolites in untargeted metabolomics remains a major challenge, particularly when relying solely on high‐resolution mass spectrometry (HRMS) data at the MS1 level. Traditional approaches using Van Krevelen diagrams often lack sufficient resolution to distinguish structurally similar metabolite classes.

**Methods:**

We developed a chemoinformatic framework that combines Van Krevelen analysis (H/C vs. O/C) with double bond equivalent (DBE) calculations to refine metabolite class annotation at Level 3 of the Metabolomics Standards Initiative (MSI). Molecular formulas were retrieved from curated structure databases and natural product repositories, and DBE values were used to refine structural classification. A dataset of over 600 curated molecular formulas representing phenolics, alkaloids, and isoprenoids was analyzed to define class‐specific patterns.

**Results:**

The combined use of DBE and Van Krevelen plots enabled improved discrimination between overlapping metabolite classes, including flavonoids, phenolic acids, coumarins, and tannins. Our framework revealed structural trends associated with aromaticity and unsaturation that are not captured by conventional MS1‐based tools. It outperforms existing Level 3 annotation strategies that rely on in silico MS/MS fragmentation or substructure matching. A case study using *Eugenia jambolana* fruit extract validated the method, revealing dominant classes such as flavonoids, phenolic acids, and tannins using only MS1 data.

**Conclusions:**

This is the first scalable framework to annotate specialized metabolites from MS1 data alone using integrated elemental ratios and structural descriptors. It enhances the annotation confidence for untargeted metabolomics, especially in complex, undercharacterized plant matrices, without requiring MS2 fragmentation.

## 1 Introduction

Accurate identification of bioactive compounds from natural sources requires comprehensive metabolic profiling. Traditional methods for isolating and characterizing metabolites remain essential in natural products research, although they can be time and resource intensive when applied to complex matrices. In contrast, untargeted metabolomics integrates advanced analytical platforms and bioinformatics to more efficiently map chemical diversity. Liquid chromatography–mass spectrometry (LC–MS) has become a cornerstone in this field due to its high sensitivity, selectivity, and throughput. Mass spectrometry (MS), in particular, enables the detection of diverse chemical species at trace levels in complex mixtures [1] and has been widely applied to characterize plant metabolomes [2, 3]. However, the large volume of MS data requires robust computational tools for metabolite annotation and biological interpretation. LC–MS/MS generates hundreds of features per sample, and bioinformatic pipelines are essential to extract meaningful chemical and biological information [4].

In LC–MS‐based metabolomics, tandem MS (MS/MS) enables the structural elucidation of ionized molecules by generating fragment ions that serve as molecular fingerprints [5]. Untargeted LC–MS/MS captures the comprehensive chemical diversity of a sample, routinely detecting hundreds of molecular features. However, the complexity of these datasets renders manual interpretation impractical. To address this, dedicated software tools have been developed to automate data processing and annotation. Structural elucidation is typically achieved through MS/MS spectral matching against experimental libraries or by in silico dereplication strategies, with molecular networking and CSI‐FingerID among the most widely used tools in plant metabolomics [6].

Metabolite annotation in untargeted metabolomics using high‐resolution MS (HRMS) is typically achieved at Confidence Levels 2 and 3, as defined by the Metabolomics Standards Initiative (MSI) guidelines. The MSI framework establishes four levels of confidence: Level 1 (confirmed identification with authentic standard and orthogonal data), Level 2 (putatively annotated compounds via library or literature matches), Level 3 (putatively characterized compound classes based on diagnostic properties), and Level 4 (unknown compounds detected only by mass spectral properties) [7].

Regarding the natural products research, platforms such as Global Natural Products Social Molecular Networking (GNPS) have significantly advanced the exploration of plant metabolomes and accelerated the discovery of bioactive compounds [8, 9]. GNPS integrates multiple in silico tools for metabolite and class‐level annotation, including Classical Molecular Networking (MN), Feature‐Based Molecular Networking (FBMN), Network Annotation Propagation (NAP), DEREPLICATOR+, Suspect Library, Moldiscovery, MS2LDA, and MolNetEnhancer [3]. Tools like CSI‐FingerID further enhance annotation by combining fragmentation tree computation and machine learning to predict molecular fingerprints [10]. These platforms enable metabolite class identification by integrating spectral patterns, recurrent substructures, and automated chemical classifications derived from molecular networks or trained models [11, 12].

Metabolomic analyses commonly utilize MS/MS acquired through data‐dependent acquisition (DDA) or data‐independent acquisition (DIA). However, the majority of metabolic features are derived from full‐scan (MS1) data, which provides 53.7% and 64.8% greater metabolome coverage compared to DIA and DDA, respectively [13]. Consequently, MS1 offers significantly broader coverage, capturing a more diverse range of metabolites in complex samples. Moreover, HRMS enables accurate molecular formula determination, facilitating in‐depth metabolome profiling in complex matrices such as phytochemical extracts. Nonetheless, chemical annotation often remains limited to Level 3, reflecting structural ambiguities. To enhance confidence in the chemical classification of features detected exclusively by MS1, we propose integrating complementary formula‐derived descriptors: Van Krevelen ratios (hydrogen‐to‐carbon [H/C] and oxygen‐to‐carbon [O/C]) and DBE values.

The use of elemental ratios and unsaturation indices to interpret HRMS data has a long tradition. The classical Van Krevelen diagram (H/C vs. O/C) was originally introduced to describe coal composition and reaction processes [14], and it has since been widely adapted to modern HRMS studies of complex mixtures. More recently, tools such as OpenVanKrevelen have demonstrated their value for the visualization of large metabolomics datasets [15]. In parallel, the aromaticity index (AI_mod) proposed by Koch and Dittmar [16] has provided a robust descriptor of unsaturation and condensed aromatic structures in natural organic matter. Together, these formula‐derived descriptors (H/C, O/C, and DBE) offer complementary chemical constraints that enrich the interpretation of HRMS data and enable more informed class‐level annotation in metabolomics.

## 2 Methodology

In this work, we introduce a methodological framework for confident, class‐level annotation of specialized metabolites, relying solely on high‐resolution MS1 data. Our approach overcomes the limitations of traditional MS2‐dependent strategies by integrating multidimensional analyses of fundamental chemical descriptors. This framework combines Van Krevelen diagrams (H/C vs. O/C atomic ratios) and DBE calculations to define distinct chemical spaces for diverse natural product classes. Validated against a curated database of over 600 compounds, this strategy provides a robust and scalable tool that enhances metabolome coverage and improves the confidence of compound classification in complex and undercharacterized biological matrices.

A comprehensive dataset of over 600 specialized metabolites was compiled through systematic mining of publicly available natural product repositories, including COCONUT, LOTUS, ChemSpider, MassBank, PubChem, and GNPS (Supporting Information). These compounds were categorized into major biosynthetic classes: phenolic compounds (e.g., flavonoids, phenolic acids, tannins, coumarins, quinones, lignans, stilbenes, and simple phenols), nitrogen‐containing metabolites (e.g., alkaloids), and isoprenoid derivatives (e.g., terpenes, steroids, and saponins).

For each compound, the molecular formula was extracted, and the H/C and O/C ratios were calculated to construct Van Krevelen diagrams. Additionally, DBE values were computed to assess structural complexity and aromatic character. Van Krevelen plots were first generated individually for each class, then overlaid to evaluate both class‐specific distribution and regions of overlap.

To define the typical compositional ranges for each class, tables were constructed showing the observed intervals of H/C and O/C values. In overlapping regions of the Van Krevelen space, DBE values were analyzed in detail using core structural scaffolds and common substitution patterns for each class. Bar plots were generated to illustrate the distribution of DBE values across metabolite classes, providing an additional axis of structural resolution that enabled discrimination between overlapping compound types. This visualization enabled clear clustering of metabolite classes based on biosynthetic origin and structural features. For instance, highly aromatic compounds such as flavonoids and coumarins clustered in regions of low H/C and high aromaticity index, whereas triterpenes and saponins occupied zones characterized by high H/C and low aromaticity index.

The integration of Van Krevelen diagrams and DBE values provided a robust framework for Level 3 annotation of specialized metabolites based solely on MS1 high‐resolution MS (HRMS) data according to the MSI guidelines [7]. This strategy effectively circumvents limitations associated with MS/MS fragmentation acquisition and in silico interpretation, offering a more inclusive and scalable approach to metabolite classification.

It is important to note that the H/C, O/C, and DBE values employed here are formula‐derived descriptors calculated from elemental compositions assigned by high‐resolution MS1 data. They are not independent experimental measurements. As such, the framework provides complementary constraints to refine class‐level annotation but does not alter the overall confidence definition. According to the MSI, our approach remains at MSI Level 3, because it enables class‐level inference but does not replace validation through MS/MS fragmentation.

## 3 Results and Discussion

In untargeted metabolomics, achieving comprehensive chemical coverage is critical for revealing the full spectrum of biological variation. High‐resolution MS1 data enable the detection and annotation of a vastly greater number of molecular features compared to MS2‐based approaches, particularly those relying on DDA or DIA, which inherently limit fragmentation to a subset of ions. By leveraging accurate mass measurements and molecular formula prediction from MS1 data, researchers can classify a broader array of metabolites, including those present in low abundance or lacking MS2 spectra. This broader metabolic insight not only enhances the structural diversity captured in the analysis but also increases the biological relevance and interpretative power of the results. As a consequence, MS1‐driven classification tools offer a more holistic and biologically meaningful representation of the metabolome, reinforcing their value in systems biology, biomarker discovery, and comparative studies across complex biological conditions. Therefore, we present here a novel strategy that enables the comprehensive and accurate characterization of a wide range of specialized metabolite classes directly from high‐resolution MS1 data.

To facilitate data interpretation, the detected metabolites were grouped into three major chemical groups: phenolic compounds (e.g., flavonoids, phenolic acids, tannins, coumarins, quinones, lignans, stilbenes, and simple phenols classes), nitrogen‐containing metabolites (e.g., alkaloids class), and isoprenoid derivatives (e.g., terpenes, steroids, and saponins classes). These groups were selected based on their biological relevance and distinct biosynthetic origins. Importantly, each class exhibits characteristic structural features such as specific functional groups, degrees of unsaturation, and elemental composition patterns that can be leveraged to achieve accurate and confident classification based solely on molecular formula data. This structure‐informed categorization enhances the reliability of metabolite annotation and strengthens the biological significance of the untargeted analysis.

### 3.1 Phenolic Compounds

**FIGURE 1:**
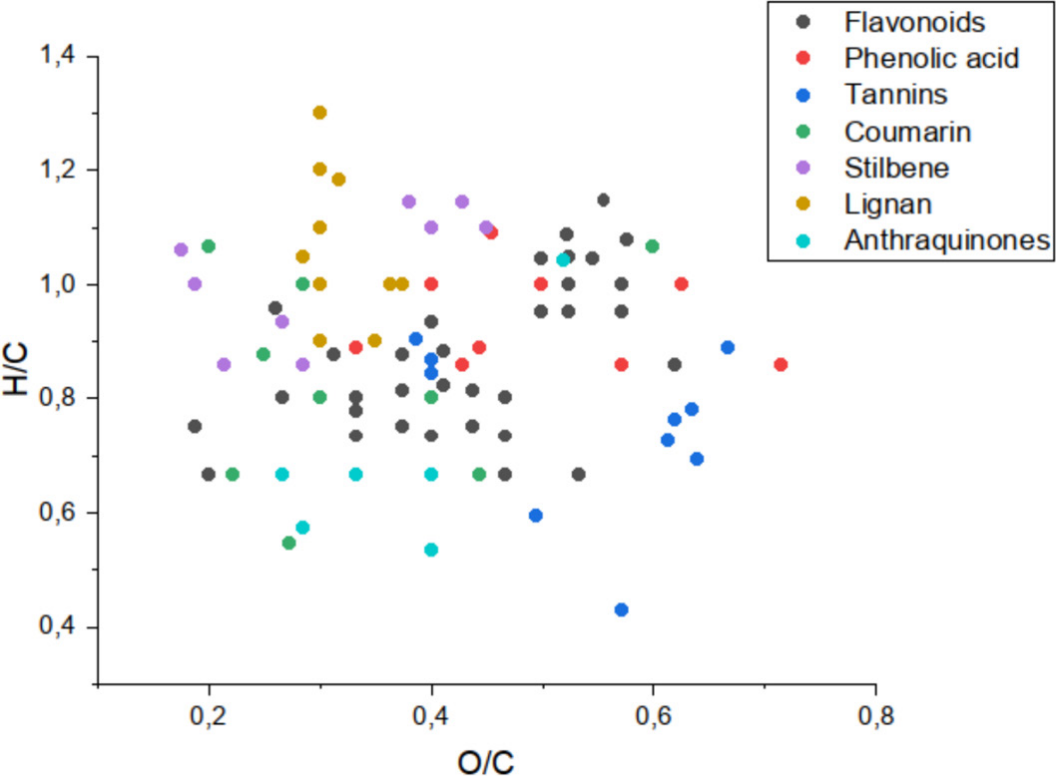
Van Krevelen diagram (H/C vs. O/C) of phenolic compounds group, illustrating class distribution based on curated molecular formulas. Each point represents a unique phenolic compound retrieved from natural product databases. Phenolic compound classes including flavonoids, phenolic acids, tannins, coumarins, stilbenes, lignans, and quinones are color coded to highlight their distinct chemical spaces.

The Van Krevelen diagram presented in Figure 1 illustrates the distribution of various phenolic compound classes based on their H/C and O/C atomic ratios, derived from 200 curated molecular formulas reported in natural product databases and literature sources, as described in Section 2. This plot provides a visual representation of the chemical space occupied by different subclasses, including flavonoids, phenolic acids, tannins, coumarins, stilbenes, lignans, and anthraquinones. Each class occupies a distinct region of the diagram, reflecting differences in structural features such as aromaticity, hydroxylation, and glycosylation. The Van Krevelen approach enables rapid discrimination of compound classes in complex mixtures, supporting the annotation and classification of phenolic metabolites in untargeted metabolomics workflows.

For flavonoids, the molecular formulas encompassed a comprehensive range of family members including anthocyanins, flavan‐3‐ols, flavonols, flavones, flavanones, isoflavones, chalcones, and rotenoids, bearing diverse substituents such as hydroxyl, methoxyl, methyl, prenyl groups, methylenedioxy bridges, and glycosyl units. For phenolic acids, the dataset included mainly benzoic acid derivatives like gallic acid and methyl gallate and cinnamic acid derivatives such as ferulic and caffeic acids. Notably, these acids exhibit numerous isomers differing only in the position of hydroxyl substituents. Tannins were represented by both condensed tannins (e.g., proanthocyanidins) and hydrolyzable tannins (e.g., gallotannins). All remaining classes (coumarins, stilbenes, lignans, and quinones) were considered in both aglycone and glycosidic forms, providing a robust chemical basis for class‐level discrimination using elemental ratio‐based analysis.

The atomic H/C and O/C ratio ranges presented for the various classes of phenolic compounds offer a powerful chemoinformatic strategy for discriminating among structurally distinct metabolite classes. These ratios, which reflect hydrogenation degree and oxidation state, respectively, are sensitive to molecular features such as hydroxylation, aromaticity, and glycosylation. For instance, flavonoids exhibit a broad O/C range (0.133–0.750) and high H/C variability (0.600–1.692), indicative of their diverse substitution patterns and degrees of unsaturation. In contrast, phenolic acids and coumarins show narrower O/C and H/C windows, reflecting their more constrained core structures. Tannins are distinguished by low H/C ratios (0.519–0.917) and moderate O/C values (0.370–0.500), consistent with their high degree of aromaticity and hydroxylation. Stilbenes, lignans, and anthraquinones occupy distinct regions in this chemical space, enabling their differentiation through Van Krevelen mapping. This elemental ratio‐based approach provides a rapid and visual means to classify phenolic compounds in untargeted metabolomics datasets, enhancing the resolution of compound annotation and structural prediction in complex plant extracts.

**FIGURE 2:**
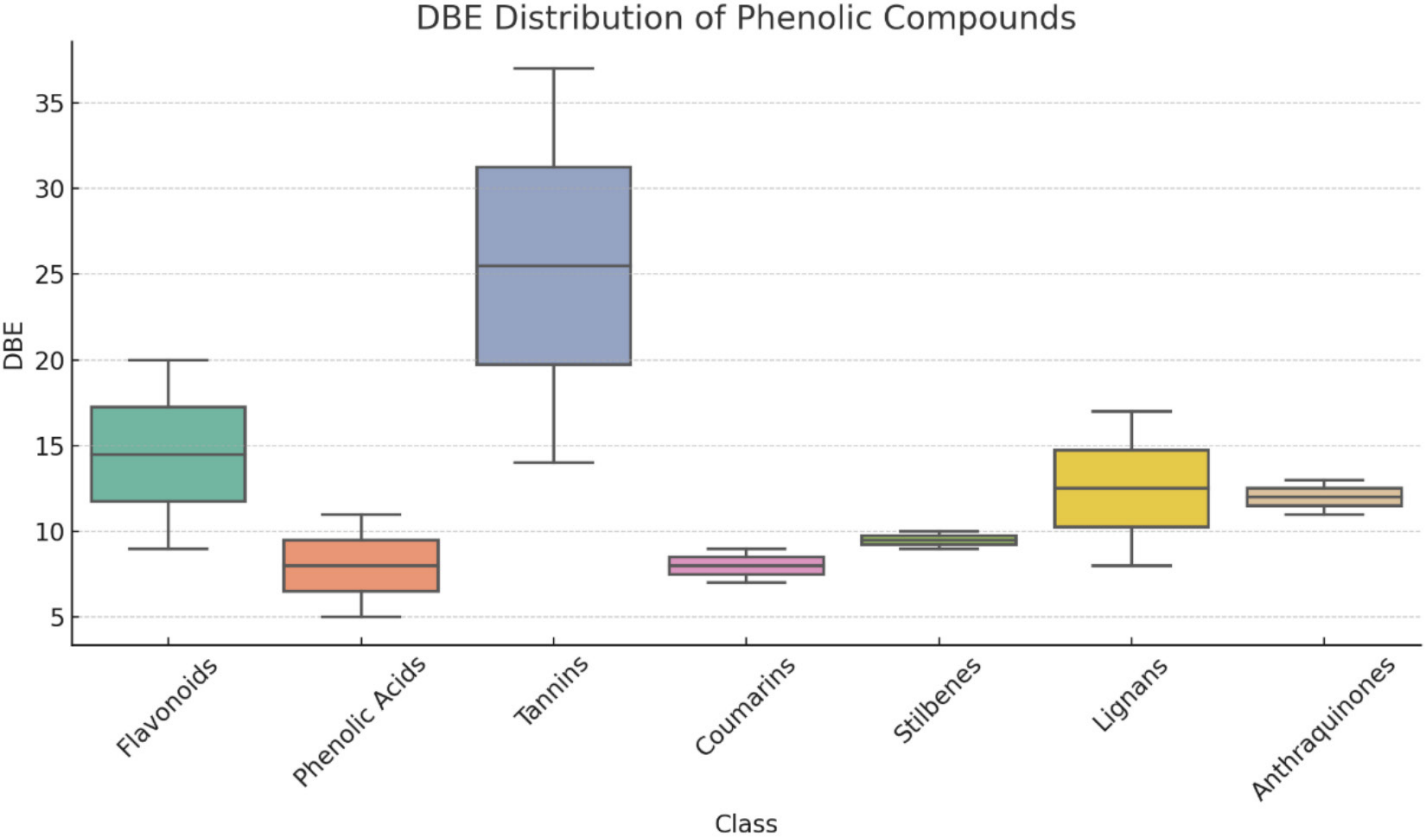
Distribution of double bond equivalent (DBE) values across different classes of phenolic compounds. The *X* axis represents DBE values, while the *Y* axis indicates the number of compounds within each class exhibiting a given DBE. Phenolic subclasses—including flavonoids, phenolic acids, tannins, coumarins, stilbenes, lignans, and quinones—are compared based on their degree of unsaturation and structural complexity. The overlapping distributions reflect variations in aromaticity and substitution patterns characteristic of each subclass.

The distribution of DBE values across different phenolic compound classes is shown in Figure 2. Among these, tannins exhibited the highest degree of unsaturation, with DBE values ranging from 14 to 37. This wide range reflects the highly polymerized nature of tannins, which consist of multiple aromatic rings and conjugated systems. In contrast, phenolic acids showed the lowest unsaturation, with DBE values between 5 and 11, characteristic of simpler aromatic structures bearing few additional unsaturations. Flavonoids, lignans, and anthraquinones presented intermediate DBE ranges. Flavonoids ranged from 9 to 20, encompassing both monohydroxylated and polyhydroxylated structures with varying levels of ring fusion and conjugation. Lignans ranged from 8 to 17, corresponding to their dimeric phenylpropanoid backbone. Anthraquinones, with DBE values between 11 and 13, exhibited relatively narrow ranges due to their conserved tricyclic aromatic core. Coumarins (DBE 7–9) and stilbenes (DBE 9–10) displayed minimal variability, likely due to their well‐defined and less‐complex structures. Despite partial overlap in DBE values between some classes, such as flavonoids and lignans or flavonoids and anthraquinones, discrimination remains feasible when considering the upper and lower DBE boundaries in combination with additional molecular features such as H/C and O/C ratios.

Overall, while some DBE ranges overlap, the maximum and minimum DBE values provide diagnostic insights that, when used in conjunction with Van Krevelen diagrams or MS/MS fragmentation patterns, contribute to a more confident classification of phenolic subclasses in complex mixtures.

### 3.2 Nitrogen‐Containing Metabolites

**FIGURE 3:**
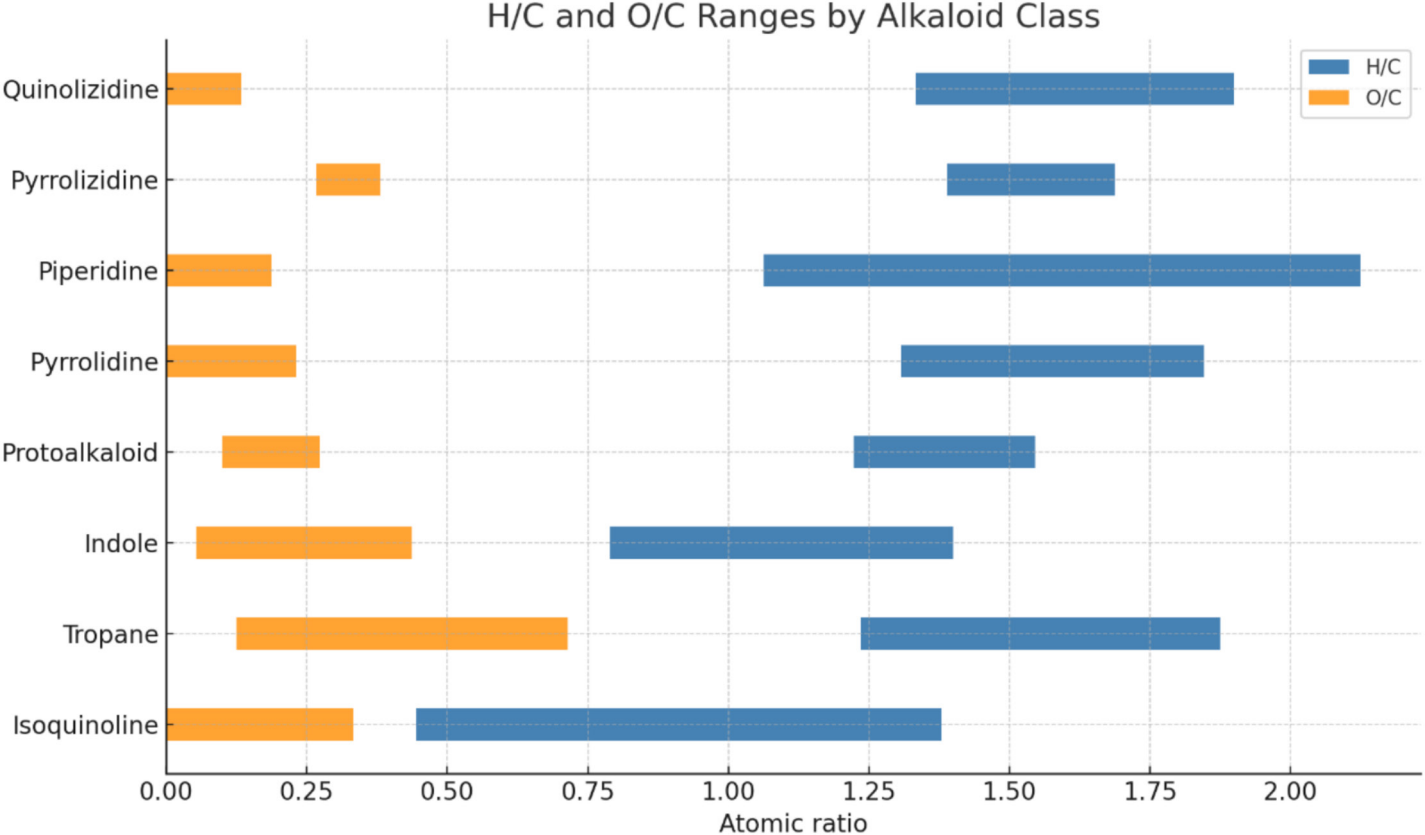
Atomic H/C and O/C ratio ranges for different alkaloid classes. The horizontal bars represent the variability in hydrogen‐to‐carbon (H/C, in blue) and oxygen‐to‐carbon (O/C, in orange) atomic ratios observed across each alkaloid class. These elemental ratios are indicative of molecular composition and oxidation state and help to distinguish between structurally and biosynthetically distinct groups such as isoquinoline, tropane, indole, and quinolizidine alkaloids.

The range plot presented in Figure 3 illustrates the distribution of various alkaloid subclasses based on their elemental ratios of H/C and O/C, derived from 300 curated molecular formulas reported in natural product databases and literature sources, as described in Section 2. This visualization reveals the structural diversity and biosynthetic distinctions across different nitrogen‐containing metabolites.

Alkaloid subclasses occupy distinct regions in the Van Krevelen space when analyzed by H/C and O/C ratio ranges (Figure 3). Isoquinolines showed the widest O/C range (0.00–0.33) and a broad H/C span (0.44–1.38), reflecting their diverse oxidation states and polycyclic aromatic scaffolds. Indole and tropane alkaloids also displayed broad intervals, consistent with their structural variability. In contrast, protoalkaloids, pyrrolizidines, and quinolizidines were confined to narrower H/C and O/C windows, suggesting more constrained frameworks. Piperidine alkaloids exhibited the widest H/C range (1.06–2.13), indicative of their aliphatic and poorly oxygenated nature. Purine and quinoline alkaloids clustered at the lowest H/C values (< 1.0), in line with highly unsaturated and aromatic structures that also show elevated DBE and AI_mod values. Collectively, these analyses demonstrate how formula‐derived descriptors (H/C, O/C, and DBE) delineate chemical boundaries among alkaloid subclasses, enabling class‐level discrimination solely from MS1‐derived molecular formula data.

**FIGURE 4:**
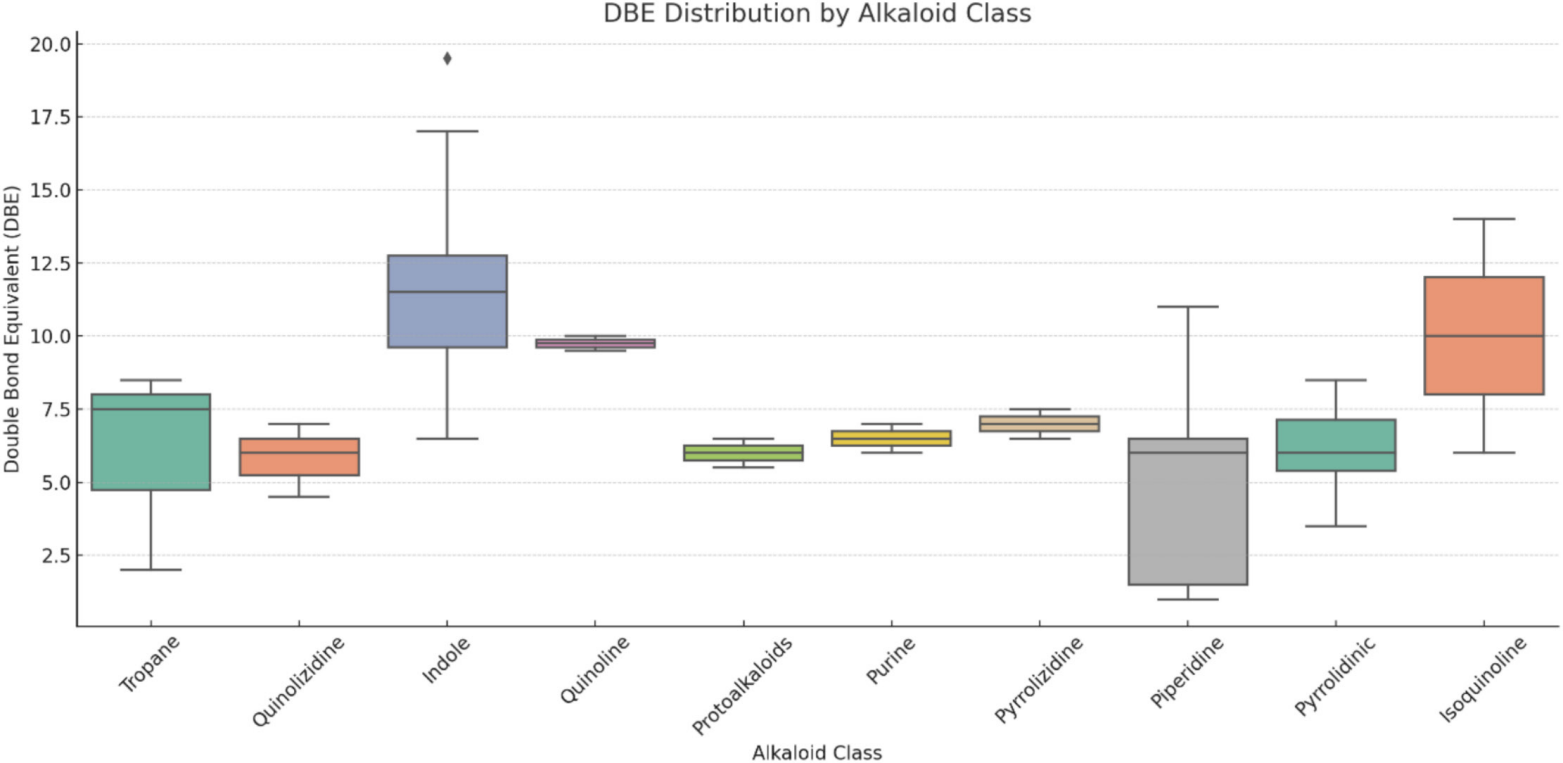
Distribution of the number of double bond equivalent (DBE) for each chemical class. The classes include indole, isoquinoline, quinoline, quinolizidine, pyridine, and tropane. The *X* axis represents DBE values, while the *Y* axis shows the number of compounds with a given DBE in each class. The overlapping distributions allow for comparison of the degree of unsaturation and structural complexity among the different chemical classes.

Although some overlap is observed, particularly among isoquinoline, indole, and protoalkaloids, the combined use of DBE and aromaticity index enables discrimination of these classes beyond what is possible with Van Krevelen plots alone. These formula‐derived descriptors provide additional constraints that refine the structural annotation of alkaloids, even without fragmentation data, thereby facilitating more reliable subclass assignments while remaining within MSI Level 3. A valuable diagnostic feature of the alkaloid class lies in the fact that most compounds contain only a single nitrogen atom in their molecular structure. Consequently, they typically exhibit a noninteger DBE value, such as 5.5 or 9.5, a distinctive characteristic not observed in any of the other compound classes analyzed. Indole alkaloids exhibited most of their DBE and modified aromaticity index values between 9 and 13, with a single compound reaching 19.5—the highest DBE observed among all alkaloid classes. The lowest DBE in this group was 6.5. Quinolizidine alkaloids showed relatively consistent DBE values ranging from 5 to 7, with only one compound presenting a DBE of 4.5. Tropane alkaloids, characterized by their bicyclic aliphatic core, predominantly exhibited a DBE of 2. However, when aromatic substituents are attached to the tropane nucleus, the DBE increases, with values ranging from 7.5 to 8.5 observed in such cases. Quinoline alkaloids displayed DBE values consistently between 9 and 10. Protoalkaloids had DBE values of 5.5 or 6.5, while purine alkaloids ranged from 6 to 7. Pyrrolizidine alkaloids presented DBE values of 6.5 or 7.5. Piperidine alkaloids, derived from a six‐membered heterocyclic amine ring, can exhibit a DBE as low as 1.5. Nonetheless, most annotated piperidine structures showed DBE values of 6 or 6.5. Pyrrolidine alkaloids had DBE values ranging from 5.5 to 8.5. The most represented class in the dataset was the isoquinoline alkaloids, with over 100 molecular formulas annotated. Although this group spanned DBE values from 6 to 14, half of the compounds clustered at DBE 10 and 11 (Figure 4).

Alkaloids as a whole constitute a highly diverse structural class, with DBE and aromaticity index values ranging from low to high. Despite this structural diversity, the biosynthesis of alkaloids is often restricted to specific plant families. As a result, the botanical origin of a sample may offer predictive insights into the likely alkaloid class present. In this context, combining molecular formula assignment with Van Krevelen diagram interpretation provides powerful diagnostic information, not only for identifying alkaloid subclasses but also for inferring structural features such as aromatic or aliphatic substituents within the annotated molecular formulas.

### 3.3 Isoprenoid Derivatives

Isoprenoid derivatives represent a chemically diverse and biologically relevant class of metabolites that can be partially annotated through the integration of Van Krevelen diagrams and DBE calculations. Due to their inherently lipophilic nature, only a subset of these compounds is detectable using electrospray ionization (ESI), which is more efficient for polar or partially oxygenated molecules. Additionally, the presence of ion suppression effects in complex matrices such as plant extracts further limits the detection of highly apolar isoprenoids. Despite these analytical challenges, triterpenes, steroids, and saponins are among the most frequently observed subclasses in high‐resolution ESI MS due to their relatively high molecular weights and the presence of oxygen‐containing functional groups that enhance ionization efficiency. Consequently, the structural interpretation of these isoprenoids can be improved through molecular formula‐based classification, enabling their distribution and chemical space to be visualized using Van Krevelen plots. In this study, 100 molecular formulas of literature‐reported ESI‐detectable isoprenoids, obtained from the databases described in Section 2, were selected to construct these visual and computational models, providing insights into their oxidation states, degrees of unsaturation, and aromatic character.

**FIGURE 5:**
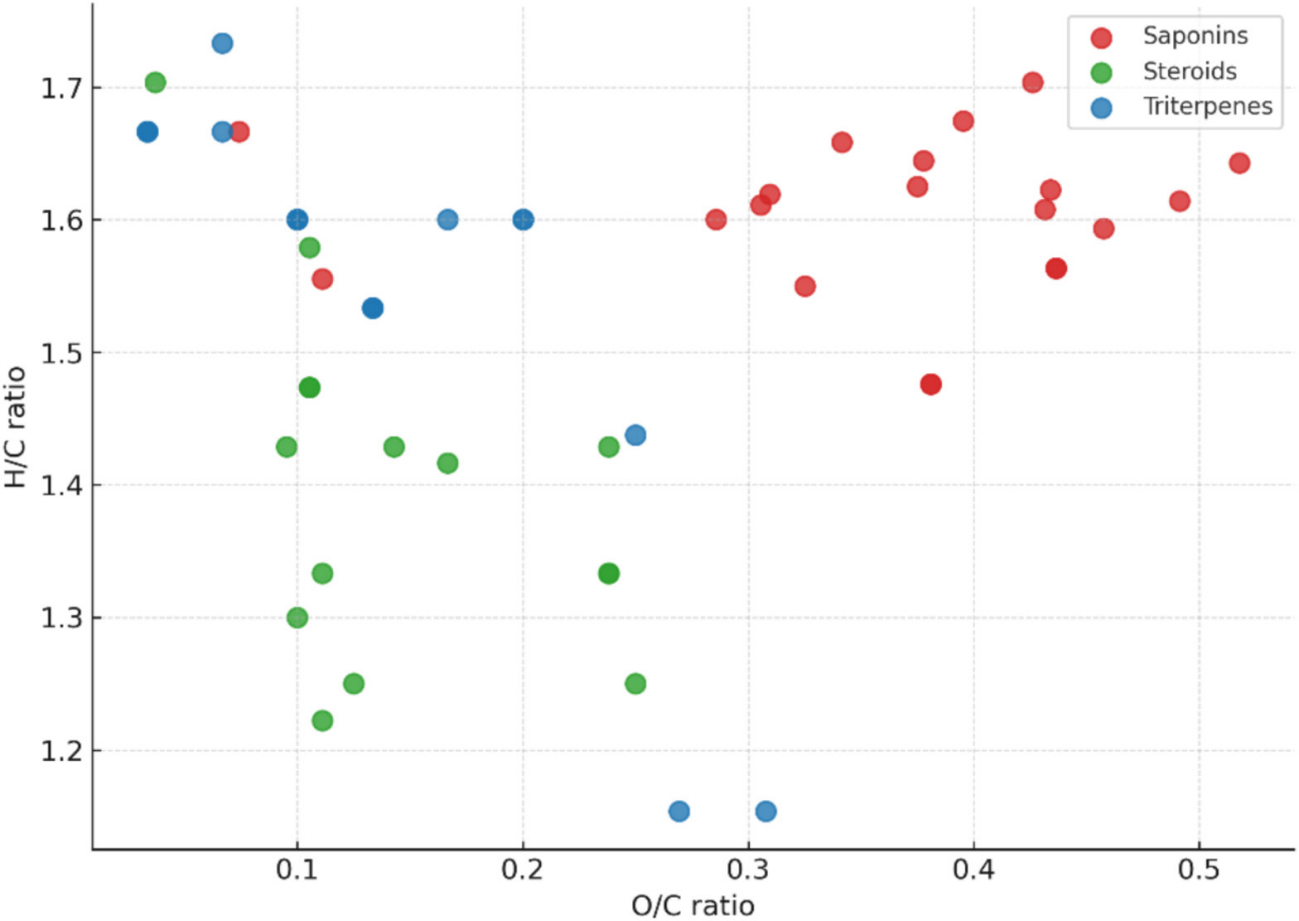
Van Krevelen diagram (H/C vs. O/C) of specialized isoprenoid derivatives (triterpenes, saponins, and steroids) showing subclass distribution based on curated molecular formulas. Each point represents a unique isoprenoid compound reported in ESI‐HRMS studies.

The Van Krevelen diagram (H/C vs. O/C) presented above reveals the chemical space occupied by isoprenoid derivatives—specifically triterpenes, steroids, and saponins—based on curated molecular formulas derived from ESI‐HRMS studies (Figure 5). Unlike phenolic compounds and alkaloids, which often exhibit overlapping Van Krevelen profiles due to their diverse substitution patterns and degrees of aromaticity, isoprenoid‐based metabolites are more distinctly separated. This is primarily attributed to their long aliphatic hydrocarbon chains and low degree of unsaturation, resulting in characteristically high H/C ratios across all three groups. Triterpenes and steroids are marked by extremely low O/C ratios, reflecting their predominantly nonpolar, oxygen‐poor structures. In contrast, saponins exhibit relatively higher O/C ratios due to the presence of glycosidic moieties (sugar units) covalently attached to the triterpenoid or steroidal core. These structural differences enable clear subclass discrimination within the Van Krevelen space, demonstrating the utility of this diagram in visualizing and annotating isoprenoid derivatives in complex mixtures.

**FIGURE 6:**
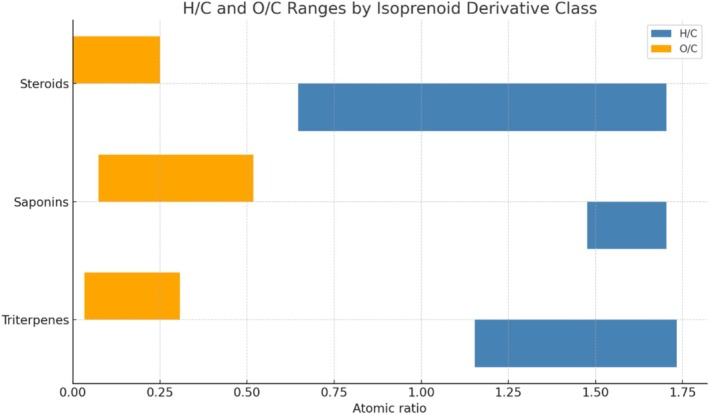
H/C and O/C atomic ratio ranges of isoprenoid derivatives (triterpenes, steroids, and saponins), based on curated molecular formulas from literature reports using ESI‐HRMS. Each bar represents the minimum and maximum values observed for each compound class.

Figure 6 illustrates the H/C and O/C atomic ratio ranges for three major subclasses of isoprenoid derivatives—triterpenes, steroids, and saponins—based on literature‐derived molecular formulas analyzed by ESI‐HRMS. Steroids exhibit H/C values ranging from 0.647 to 1.704 and O/C values between 0.000 and 0.250, reflecting their predominantly aliphatic structure with minimal oxygenation. Triterpenes also show high H/C values (1.154–1.733) and slightly higher O/C values (0.033–0.308), indicative of their similar hydrocarbon backbone but with modest oxygen incorporation. In contrast, saponins are clearly distinguished by their elevated O/C ratios (0.074–0.518), due to the presence of glycosidic units, while maintaining high H/C values (1.476–1.704), consistent with their amphiphilic nature and enhanced ionization in ESI conditions.

Triterpenes are organic compounds characterized by their structure consisting of four or five rings and one or more double bonds. Due to these structural features, the degree of unsaturation (DBE) values for triterpenes are generally higher than 5. In our findings, the calculated DBE for these triterpenes ranges from 5 to 12. The most frequent DBE value was 6, suggesting a common structural motif involving five rings and double bonds within the framework. The higher DBE values, reaching up to 12, indicate the presence of five rings and multiple double bonds, likely due to the incorporation of unsaturations such as carbonyls or conjugated double bonds, beyond the typical pentacyclic or tetracyclic triterpene structure with some double bonds.

Steroids exhibited DBE values similar to those of triterpenes, ranging from 5 to 10. While triterpenes typically contain 30 carbon atoms, derived from six isoprene units, steroids possess a closely related carbon framework but generally with a lower number of carbon atoms. Structurally, steroids are usually composed of four rings and varying degrees of unsaturation. Despite the overlap observed in Van Krevelen diagrams and DBE values between steroids and triterpenes, the total number of carbon atoms can serve as a diagnostic criterion to distinguish between these two classes. Additionally, saponins showed DBE values ranging from 7 to 13, with a predominant DBE of 8. This increased degree of unsaturation is primarily attributed to additional ring structures introduced by sugar moieties covalently linked to the polycyclic backbone of either triterpenes or steroids. These glycosidic units contribute to the overall unsaturation by adding cyclic structures, which explains the higher DBE values observed in comparison to their aglycone precursors.

**FIGURE 7:**
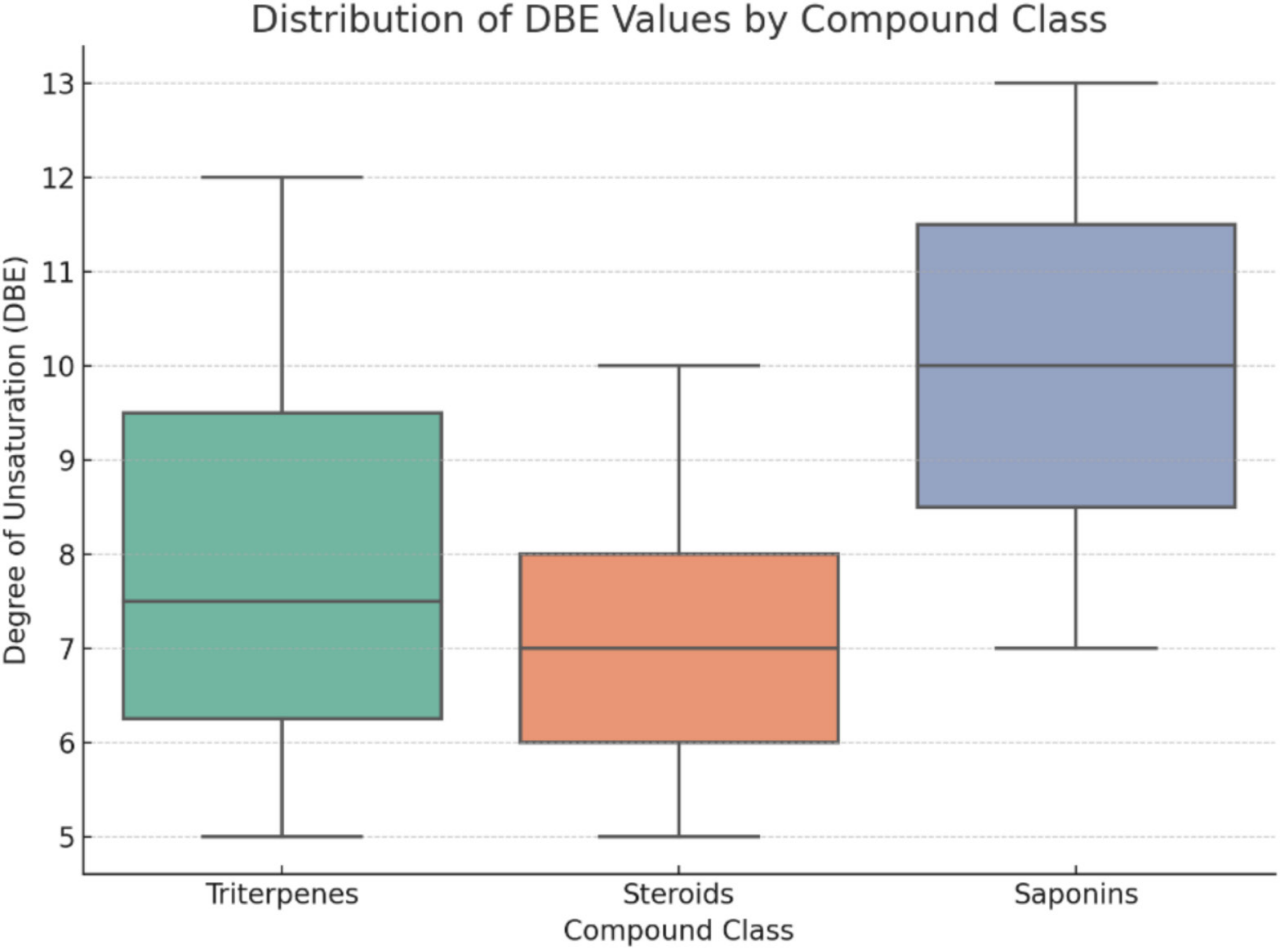
Boxplot showing the distribution of DBE values for triterpenes, steroids, and saponins. While triterpenes and steroids exhibit overlapping DBE ranges, saponins show higher values due to additional sugar units, highlighting DBE as a structural indicator rather than a definitive classification tool.

Although the DBE values alone were not effective in fully distinguishing between the three classes (saponins, steroids, and triterpenes), they provided valuable insights into the degree of unsaturation and the presence of sugar moieties attached to the polycyclic core structures. The overlap in DBE ranges reflects the structural similarities among these compounds, particularly between triterpenes and steroids (Figure 7). However, the Van Krevelen diagram proved to be a more effective tool for class separation, allowing for clearer differentiation based on elemental composition and functional group distribution.

**FIGURE 8:**
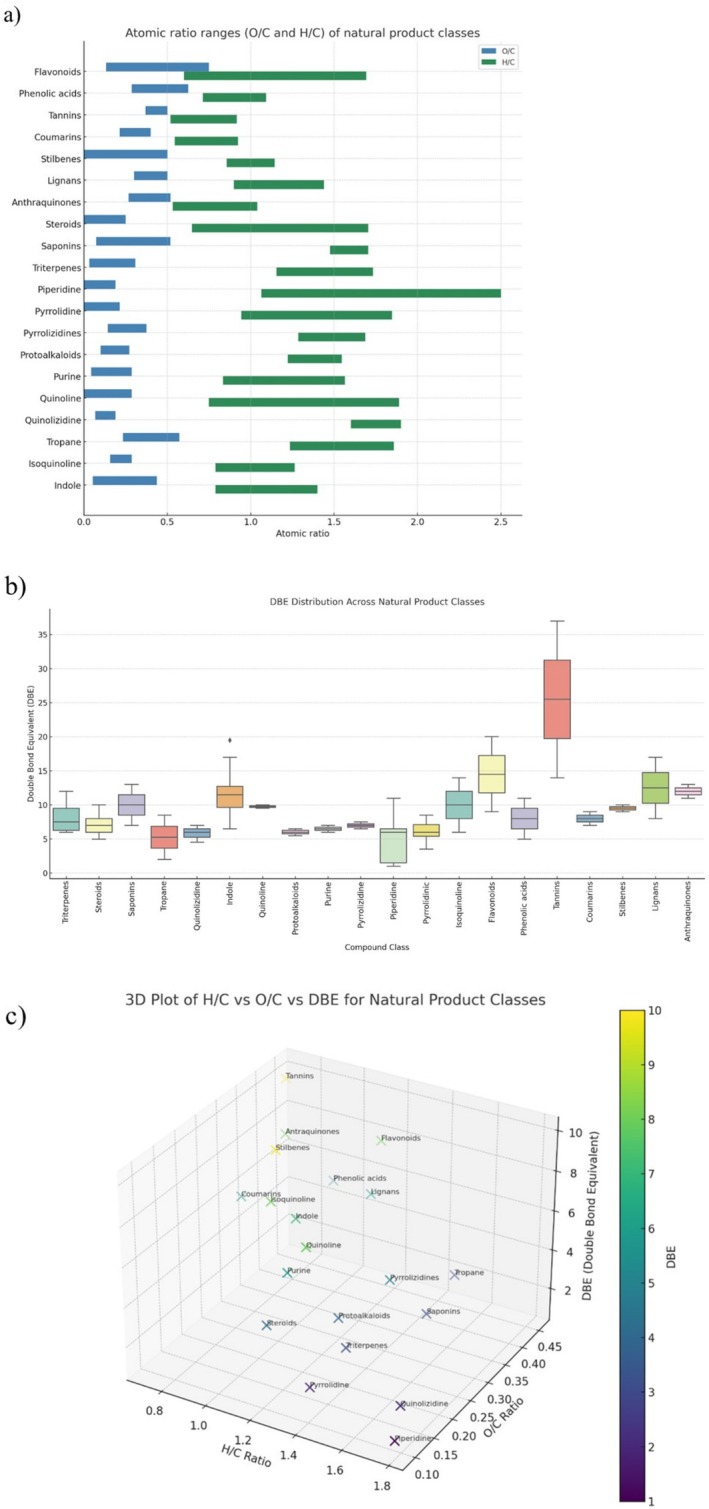
(a) H/C and O/C atomic ratio ranges for major natural product classes based on curated molecular formulas from literature using ESI‐HRMS. Classes include isoprenoid derivatives (triterpenes, steroids, and saponins), nitrogen‐containing compounds (isoquinoline, indole, tropane, piperidine, pyrrolidine, quinolizidine, purine, quinoline, protoalkaloids, and amino acid–derived alkaloids), and phenolic compounds (flavonoids, phenolic acids, tannins, quinones, stilbenes, coumarins, and lignans). Each bar represents the observed minimum and maximum atomic ratio for each class. (b) Boxplot showing the distribution of DBE values for the same compound classes, highlighting structural variability within and across the three major groups. (c) Three‐dimensional plot illustrating the distribution of natural compound classes based on their hydrogen‐to‐carbon (H/C), oxygen‐to‐carbon (O/C) atomic ratios, and double bond equivalents (DBEs).

To provide a more comprehensive comparative overview, the three major groups of natural products—isoprenoid derivatives, nitrogen‐containing compounds, and phenolic compounds—were integrated into unified visual representations (Figure 8). Unlike earlier sections where these groups were analyzed separately, the combined figures allow for a clearer assessment of their physicochemical diversity. Specifically, Figure 8a presents the ranges of H/C and O/C atomic ratios across all classes, while Figure 8b summarizes the DBE distributions. Figure 8c provides a three‐dimensional representation of the same data, combining H/C, O/C, and DBE values to illustrate the overlap and clustering among compound classes. This integrated approach facilitates the identification of overlapping characteristics and distinct structural patterns among the major natural product families.

While the atomic ratios of H/C and O/C offer valuable insights into molecular composition, they alone may be insufficient to distinguish between plant compound classes that occupy overlapping chemical spaces. When two or more classes exhibit significant overlap in both H/C and O/C axes, their discrimination becomes increasingly challenging, particularly when the relative intersection of their ranges is extensive. Although no compound classes in the present dataset show complete overlap, several exhibit partial convergence that may hinder classification. For instance, flavonoids, phenolic acids, and lignans share closely aligned ranges in both O/C and H/C, while pyrrolidine and quinoline demonstrate similar values, particularly in the O/C range (0.0 to ~0.2–0.3) and H/C (0.9–1.8). These overlaps can be quantified using interval intersections across axes, highlighting compound groups such as flavonoids versus phenolic acids, lignans versus anthraquinones, and piperidine versus pyrrolidine versus pyrrolizidines versus protoalkaloids. Similarly, classes like triterpenes, saponins, and steroids also exhibit substantial intersection in atomic ratios, suggesting the need for additional molecular descriptors. In this context, the DBE emerges as a powerful discriminative parameter. DBE captures the degree of unsaturation and aromaticity—structural features intrinsically linked to chemical classification. Compounds sharing similar O/C and H/C values can exhibit dramatically different DBE values. For example, a flavonoid and a saponin may both possess H/C ~ 1.5 and O/C ~ 0.4, yet flavonoids typically feature highly conjugated aromatic rings (DBE ≥ 8), whereas saponins are glycosylated triterpenoids with lower DBE values (< 6). Likewise, alkaloids such as piperidine (saturated, DBE = 1) contrast sharply with aromatic systems like quinoline or isoquinoline (DBE ≥ 7), despite overlapping atomic ratios. Even among structurally related groups—such as tannins, phenolic acids, and lignans—the DBE adds critical separation, with tannins (highly polymerized phenolics) exhibiting substantially higher unsaturation levels. The 3D plot (Figure 8c), integrating DBE with O/C and H/C ratios, adds a structural dimension to the classification framework. This approach enables clearer separation between aromatic and saturated compounds, enhances discrimination of alkaloid subtypes, and reveals distinctions between molecules with similar atomic compositions but fundamentally different architectures.

In addition to double bond equivalence, the number of carbon atoms can serve as a decisive parameter for distinguishing between natural product classes. Two‐dimensional plots such as DBE versus carbon number provide valuable insights into molecular diversity and structural complexity. For instance, flavonoids predominantly cluster within the range of 15–25 carbon atoms and exhibit DBE values between 7 and 17, consistent with their aromatic polyphenolic structures. Phenolic acids, on the other hand, are characterized by lower carbon content (7–11) and relatively low DBE values (5–7), reflecting simpler aromatic frameworks. Tannins display a wide range of carbon atoms (30 to over 100) and elevated DBE values, indicative of highly unsaturated and polymerized structures. Triterpenes consistently contain 30 carbon atoms, while saponins exhibit higher carbon counts (35–60) due to glycosidic sugar moieties. Steroids typically possess fewer than 29 carbon atoms. These trends underscore the utility of DBE versus carbon number plots as a powerful chemotaxonomic tool for supporting class discrimination in complex metabolomic datasets.

Given that a plant extract contains hundreds of metabolites, and HRMS is capable of resolving these hundreds of metabolites and assigning molecular formulas to the detected species, the combination of these tools can be extremely valuable for a more nuanced and detailed analysis.

## 4 Case Study and Visualization

We demonstrate the applicability of this framework using an untargeted metabolomics dataset derived from a medicinal plant extract. Fresh fruits of *Eugenia jambolana* were extracted at room temperature using a hydroalcoholic solution consisting of 70% ethanol and water under continuous agitation. MS1 analyses from crude extract were performed on a Q‐Exactive Hybrid Quadrupole‐Orbitrap high‐resolution mass spectrometer (Thermo Scientific), equipped with an ESI source operating in positive ionization mode. Molecular formulas were generated using Thermo Scientific Xcalibur software based on accurate mass measurements and isotopic patterns. Molecular formulas with mass errors less than 2 ppm were considered. The resulting dataset was subsequently explored using Van Krevelen diagrams (H/C vs. O/C atomic ratios) and DBE distribution plots to facilitate chemical classification and structural inference.

Cluster patterns matched known phytochemical classes, validating the approach. Importantly, the combination of DBE values and carbon number enabled the differentiation of metabolic classes that overlap in the Van Krevelen diagram alone. Furthermore, the proposed approach enables a more comprehensive and inclusive assessment of metabolites, as it relies solely on MS1 HRMS data and circumvents the bottlenecks associated with acquiring fragmentation spectra.

**FIGURE 9:**
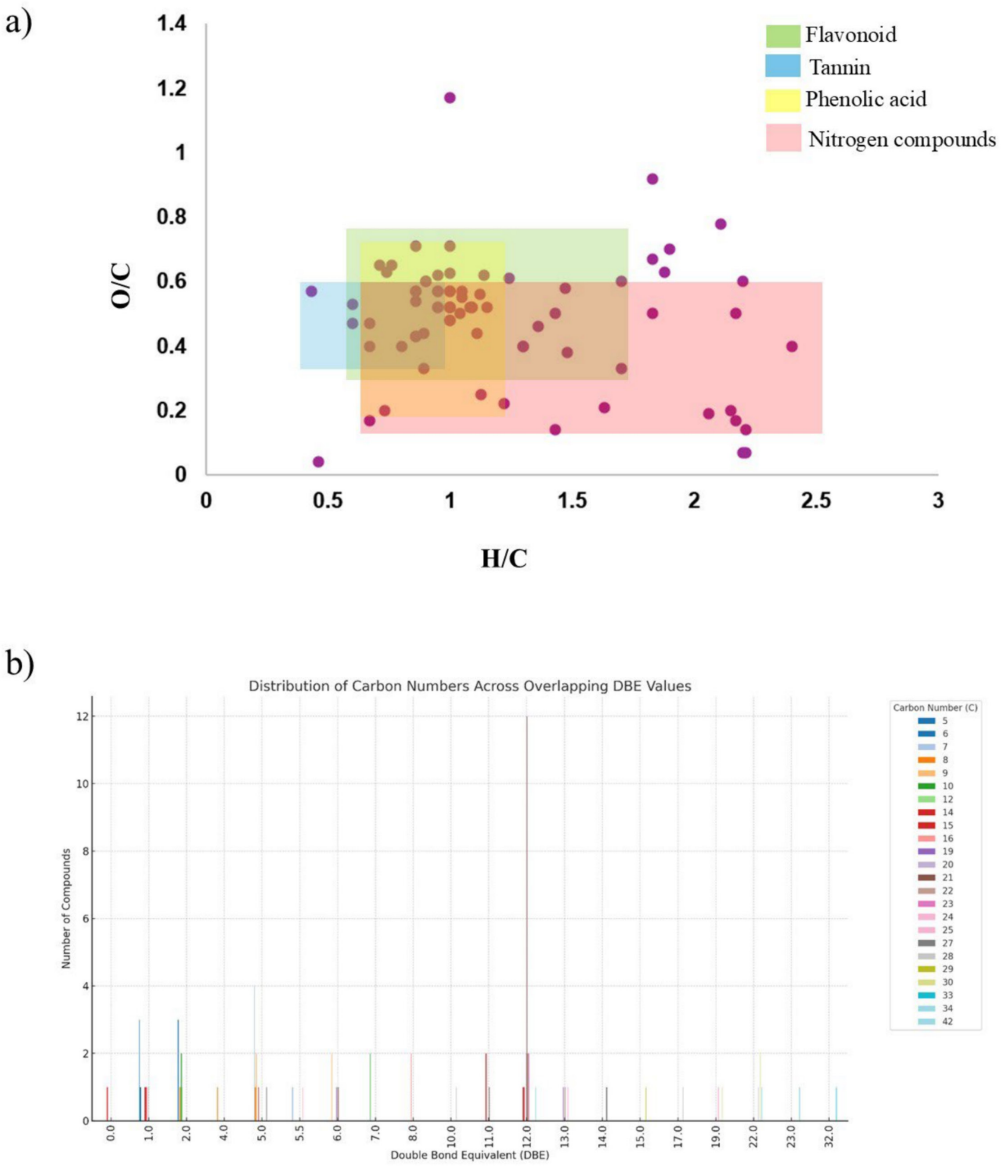
Van Krevelen diagram (a) and DBE distribution plot (b) of fruit extracts from *Eugenia jambolana* . The Van Krevelen diagram displays the relationship between the oxygen‐to‐carbon (O/C) and hydrogen‐to‐carbon (H/C) atomic ratios for the detected metabolites, calculated from molecular formulas obtained via high‐resolution Orbitrap mass spectrometry (HRMS).

The Van Krevelen diagram (Figure 9) revealed a prominent distribution of phenolic compounds and other polar metabolites, which is consistent with the chemical nature of both the extraction solvent and the fruit matrix. The use of a hydroalcoholic solvent favored the selective extraction of oxygen‐rich, polar compounds, resulting in a metabolite profile dominated by species with high O/C and variable H/C ratios, characteristic of phenolics and related secondary metabolites.

A significant number of molecular formulas were assigned to primary metabolites such as sugars, aliphatic and aromatic amino acids, lipids, and ribonucleosides. However, the primary focus of this study is the classification and characterization of secondary metabolite classes. A comprehensive review of the literature on the chemical composition of *E. jambolana* fruit indicates that the polar extracts are predominantly composed of phenolic compounds including both aglycone and glycosylated flavonoids, phenolic acids, tannins, and saponins [17, 18].

The DBE distribution plot (Figure 9b) reveals a high abundance of compounds with DBE values of 11 and 12, which can be primarily attributed to glycosylated flavonoids such as anthocyanins, pigments widely distributed in the peel and pulp of *E. jambolana* fruits. This interpretation is further supported by the Van Krevelen diagram (Figure 9a), where these compounds appear in regions with high O/C ratios (~0.5) and intermediate H/C values (~0.9), consistent with aromatic structures bearing sugar moieties. Additionally, the presence of phenolic acids was identified, as these compounds exhibit characteristic H/C ratios indicative of aromatic frameworks (~0.9), intermediate O/C values (~0.4) derived from hydroxyl groups, a low carbon number, and DBE values around 5–6, features typical of structures composed of an aromatic ring and a carboxylic group. On the other hand, a large number of nitrogen‐containing compounds were detected, most likely corresponding to amino acids and other derived primary metabolites, because the occurrence of alkaloids has rarely been reported within the genus *Eugenia*.

Tannins were also detected based on their distinct oxygenation pattern in the Van Krevelen space, defined by high O/C ratios and a strongly aromatic character (low H/C ratio), in conjunction with notably high DBE values (e.g., DBE = 19). Furthermore, the presence of compounds with both high O/C and H/C ratios and intermediate DBE values (e.g., 7–8) suggests the occurrence of glycosylated aliphatic structures, such as saponins. A significant number of nitrogen‐containing compounds were also observed; however, these are most likely derived from amino acids or nonalkaloidal nitrogenous compounds.

The combined use of DBE distribution analysis and Van Krevelen diagrams, when integrated with prior knowledge of the studied species, enables a more accurate inference of specialized metabolite classes while allowing for the exclusion of unlikely compound types. This synergistic approach enhances the reliability of molecular characterization by aligning chemical patterns with known phytochemical profiles.

## 5 Conclusion

This study introduces and validates a comprehensive strategy for Level 3 metabolite annotation based exclusively on high‐resolution MS1 data, combining DBE distribution with Van Krevelen diagram analysis. Using *E. jambolana* as a case study, we demonstrate the power of this approach to assign major classes of specialized metabolites—without the need for MS2 fragmentation—by leveraging fundamental chemical descriptors derived from accurate molecular formulas. Importantly, this strategy encompasses the main classes of secondary metabolites typically ionized by ESI, including phenolic compounds (e.g., flavonoids, phenolic acids, tannins, coumarins, quinones, lignans, stilbenes, and simple phenols), nitrogen‐containing metabolites (e.g., alkaloids), and isoprenoid derivatives (e.g., terpenes, steroids, and saponins). Given that MS2 spectra are acquired for only a fraction of MS1 features in untargeted analyses, this MS1‐based annotation method offers broader metabolome coverage and enhanced applicability, particularly for nonmodel species and complex plant matrices. Overall, the integration of DBE and Van Krevelen representations provides a scalable and chemically informed framework to support class‐level metabolite annotation in untargeted metabolomics workflows.

## Author Contributions

**Nerilson M. Lima:** conceptualization, investigation, writing – original draft, methodology, validation, visualization, writing – review and editing, supervision, resources, formal analysis. **Luana A. Pereira:** conceptualization, investigation, writing – original draft, methodology. **Lucas S. Tironi:** conceptualization, investigation, writing – original draft, methodology. **Matheus P. G. do Carmo:** conceptualization, investigation, writing – original draft, methodology. **Milbya L. Costa:** conceptualization, investigation, writing – original draft, methodology. **Renato A. Oliveira:** conceptualization, investigation, writing – original draft, methodology. **Salva Asghar:** conceptualization, investigation, writing – original draft, methodology. **Vinicius Fortes da Silva:** conceptualization, investigation, writing – original draft, methodology.

## Ethics Statement

The authors have nothing to report.

## Conflicts of Interest

The authors declare no conflicts of interest.

## Peer Review

The peer review history for this article is available at https://www.webofscience.com/api/gateway/wos/peer‐review/10.1002/rcm.10145.

## Supporting information




**Data S1:** Supporting Information.

## Data Availability

The data that support the findings of this study are available from the corresponding author upon reasonable request.
